# Social and Poor Law Problems

**Published:** 1907-01-26

**Authors:** 


### SOCIAL AND POOR LAW PROBLEMS.

### THE CONSOLIDATION OF THE POOR-LAWS.

"At the present time the Acts of Parliament affecting the Poor-laws of England alone, exclusive of Scotland and Ireland, number upwards of 130." This statement, which is quoted from the "Encyclopædia Britannica," and may therefore be taken as fairly authoritative, may well give pause to the people who are always exclaiming at the Poor-law and the way in which it is administered. It is practically impossible for those who administer these laws to have at their finger-ends a working knowledge of all of them, and, as a matter of fact, many Guardians have but the vaguest knowledge of them, and have to be pulled up continually by the officials, who are nominally their servants, when, either in the direction of laxity or severity, they propose to act in opposition to the law. Not that the officials understand more than their own little corner of the Acts—those which affect their personal work. And regarding almost every point one can think of, there is so much uncertainty that constant appeals for guidance are made to the Local Government Board—appeals which are answered sometimes according to strict legality, but sometimes, when the problem presented is a new one, according only to the judgment of those in authority. In spite of all the laws, cases arise for which at present no provision is made. Thus, recently, it was proved that a married woman, even though possessed of private means, cannot be compelled to maintain her parents. As the vast majority of married women whose parents are likely to come on the rates have nothing but what they get from their husbands, the necessity of making arrangements for such an unlikely contingency had never presented itself to the lawmakers, yet now it comes up for settlement. The arrangements for the feeding of school children have caused endless trouble and uncertainty as to what is legal and what is not. And one could go on indefinitely pointing out difficulties which our present Acts do not deal with. On the other hand, regulations appear on the statute book which have long ceased to be of general utility, and may on occasion operate very harshly. That a final and complete Poor-law can be framed, which will never require revision, is not possible. The development of civilisation will bring new problems to each generation. But this does not affect the fact that if our present Poor-laws were revised and consolidated, unnecessary regulations and such provisions as contradict each other being left out, it might be more easy than it is now for the plain man who is a Guardian to grasp the principles of the law he is administering. In time new accretions would doubtless gather, but that is no reason for not removing the present accumulation of barnacles. This is a reform which has long been demanded by students of the Poor-law, and very persistently by Mr. H. R. Gawen Gogay, formerly a member of the St. Saviour's Board of Guardians. Having had to deal with the complications of our present laws, he knows the need for reform, and it is to be hoped that he will be able to persuade other Guardians to press the matter forward in such a way that the administration of these laws may be made easier, if not for them at least for those who come after them. The existing difficulty of forming a clear idea of what the law demands and permits, forms a constant source of trouble to Guardians, causes irritation to officials, and not seldom hardship to the poor.

Mr. J. Stroud Hosford, F.R.C.S.(Ed.), has been appointed honorary assistant surgeon to the Royal Eye Hospital, Southwood.

